# Successful Flexible Bronchoscopic Management of Dynamic Central Airway Obstruction by a Large Tracheal Carcinoid Tumor

**DOI:** 10.1155/2014/349707

**Published:** 2014-11-13

**Authors:** Vijay Hadda, Karan Madan, Anant Mohan, Umasankar Kalai, Randeep Guleria

**Affiliations:** Department of Pulmonary Medicine and Sleep Disorders, All India Institute of Medical Sciences, New Delhi 110029, India

## Abstract

Typical carcinoid of the trachea presenting as an endoluminal polypoidal mass is a rare occurrence. Herein, we report a case of a 34-year-old female patient who presented with features of central airway obstruction. Flexible bronchoscopy demonstrated a large pedunculated growth arising from the lower end of the trachea near carina which was flopping in and out of the main tracheal lumen and the proximal right bronchus leading to dynamic airway obstruction. Successful electrosurgical excision (using a snare loop) of the polypoidal growth was performed using the flexible bronchoscope itself. The patient had immediate relief of airway obstruction and histopathological examination of the polyp demonstrated features of typical carcinoid (WHO Grade I neuroendocrine tumor).

## 1. Introduction

Endobronchial benign tumors of the respiratory tract are rare [1, 2]. Carcinoids are among the commonest endobronchial benign tumors; however, primary typical carcinoids of the trachea are uncommon [1, 2]. Due to slow growth rate, symptoms are often mistaken for bronchial asthma and a delay in diagnosis is usual. Hemoptysis may not be present in all the patients. Trepopnea is an underrecognized form of dyspnea where patient is having breathing difficulty in only one lateral decubitus position. We report a case of a young female patient who presented with trepopnea and with a normal chest X-ray examination. CT scan followed by a bronchoscopic examination of the airways demonstrated the presence of a large pedunculated tracheal tumor which was successfully removed with endobronchial electrocautery with a snare loop using flexible bronchoscopy. Primary tracheal tumors are rare neoplasms which can be missed due to paucity of symptoms and difficulty in detecting them with chest radiographs.

## 2. Case Presentation

A 34-year-old lady presented with history of shortness of breath of four-month duration and shortness of breath used to be worse on lying in the left lateral position (trepopnea). She also complained of dull aching central chest pain and dry cough for the same duration. In addition, she also complained of recurrent seasonal episodes of sneezing and nasal obstruction associated with headache. There was no history of fever, wheeze, and hemoptysis or weight loss. General physical examination revealed expiratory stridor. Respiratory rate was 24/min and oxygen saturation while breathing at room air was 95%. Examination of the respiratory system and rest of the systemic examination was normal.

**Figure 1:**
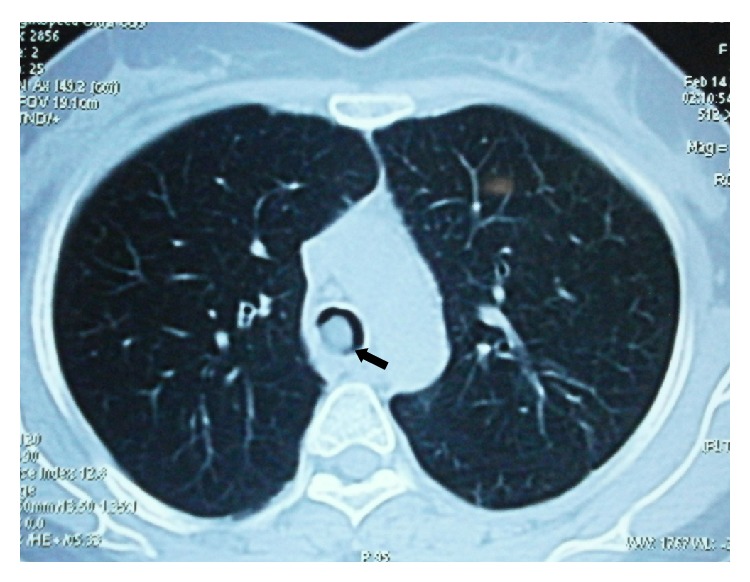
CT scan of the thorax showing a endoluminal polypoidal growth (arrow) arising from the posterior wall of the tracheal bifurcation.

Chest radiograph was normal. In a young female in view of trepopnea and expiratory stridor, intrathoracic (tracheal/major bronchial) obstruction was suspected and contrast enhanced computed tomography (CT) scan of the thorax was performed. In view of symptoms suggestive rhinosinusitis CT of paranasal sinuses was also performed. CT scan of the thorax showed presence of endoluminal polypoidal growth arising from the posterior wall of the tracheal bifurcation measuring 13 mm in anteroposterior diameter and 13 mm in the transverse diameter (Figure 1). The growth was not enhancing postcontrast administration. There was no mediastinal lymph node enlargement and both lungs appeared normal.

**Figure 2:**
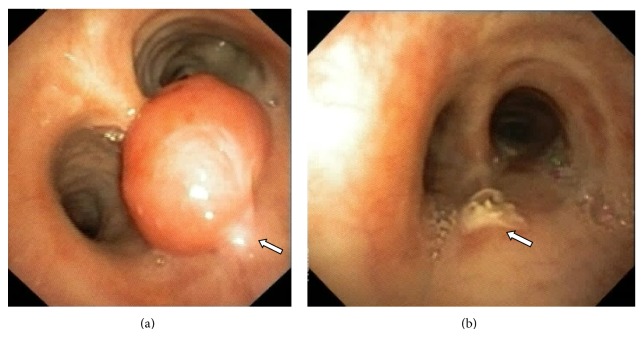
(a) Flexible bronchoscopic image showing a large fleshy polypoidal growth with well-defined narrow stalk (arrow) arising from the posterior wall of the lower trachea. (b) The electrosurgical excision successfully removed the tumor in toto (b). There was no bleed or gross evidence of residual tumor at the intervention site (arrow).

Flexible fiberoptic bronchoscopy (FOB) was performed for further assessment. It revealed a large fleshy polypoidal growth with well-defined narrow stalk arising from the posterior wall of the lower trachea (Figure 2(a)). The growth was flopping in and out of the right main bronchus and trachea leading to dynamic airway obstruction of either of the two main bronchi. The major bronchi and the distal bronchial segments were normal.

In view of the respiratory distress (central airway obstruction), a decision to attempt flexible bronchoscopic removal of the polyp was taken. Facility for rigid bronchoscopy was standby, in case there was any difficulty during flexible bronchoscopic removal. Olympus BF-1T-180 video bronchoscope (with a 3 mm working channel) via oral route was used. An electrosurgical snare loop (SD 7C-1, Olympus) was used for electrosurgical excision. The snare loop was used to encircle and firmly tighten around the neck of the polypoidal growth and electrosurgical excision was performed. The mass got avulsed from its base completely and stuck on to the snare loop and the same was removed in toto along with the flexible bronchoscope (Figure 2(b)). The procedure was done under conscious sedation using lidocaine nebulization and intravenous injection of midazolam 2 mg, fentanyl 50 microgram, and Phenergan (promethazine) 25 mg. There was no procedural complication/bleeding. Patient had immediate relief of symptoms of breathlessness and trepopnea in the same day.

**Figure 3:**
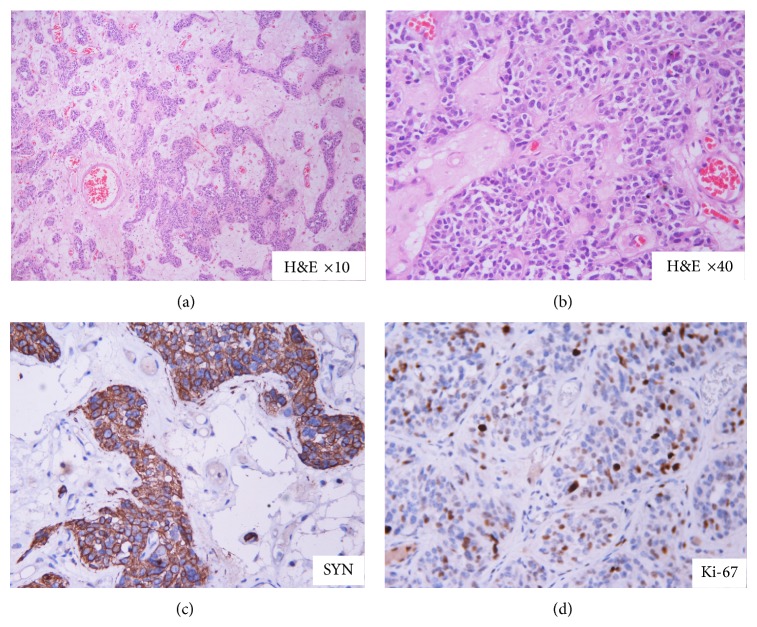
Histopathology of the ablated tumor showing features of carcinoid. Tumor cells are arranged in nests with rich vascular stroma (a). They show salt and pepper type of nuclear chromatin (b). Immunohistochemistry shows positivity for synaptophysin (c) with increased proliferating (KI 67 labelling) index (d).

The histopathology of the tracheal growth was suggestive of carcinoid (Figure 3). A DOTANOC Positron Emission Tomographic CT scan was performed later which demonstrated no areas of abnormal tracer uptake. Patient is under regular follow-up and follow-up bronchoscopic examination has been normal.

## 3. Discussion

Carcinoids arise from the Kulchitsky cells disseminated in the bronchopulmonary mucosa [1]. These tumors are uncommon and account for approximately 2% of all bronchopulmonary tumors [3, 4]. Most of bronchopulmonary carcinoids (75–90%) are localized to central airways whereas smaller proportion (10–25%) is peripheral [3, 4]. According to WHO 2004, neuroendocrine tumors of the lung are divided into three main entities: carcinoid tumors (typical/atypical), large cell neuroendocrine carcinomas (LCNEC), and small cell carcinomas (SCC) [5]. These are further classified as well differentiated (low grade) typical, moderately differentiated (intermediate grade) atypical, and poorly differentiated (high grade) LCNEC and SCC based on the appearance, mitotic rate, Ki-67 index, and presence of necrosis [5].

Typical carcinoid tumors, which represent 90% of carcinoid lung neoplasms, occur mostly in young patients. Among patients with typical carcinoids metastases to lymph node (5%–15%) and distant sites (3%) are uncommon at presentation. In comparison, atypical carcinoids though rare (0.1%-0.2%) lung tumors, however, lymph node (40%–50%) or distant (20%) metastasis are common at presentation [6]. Bagheri et al. reported that the most common site of involvement was the left main bronchus (25%) while tracheal involvement was seen in 5% [7]. Tracheal carcinoids most commonly arise from the distal one third of the trachea from the posterior noncartilaginous fibrous membrane as in our case. The usual symptoms are dyspnea, wheezing, and hemoptysis; however, our patient presented with unusual symptom of trepopnea which has never been reported in the literature previously. Trachea being a blind spot in the chest radiograph and the symptoms of wheeze and stridor are misinterpreted as asthma; there remains a delay in the diagnosis of primary tracheal tumor. 68Ga-DOTATOC PET/CT is a useful imaging investigation for the evaluation of pulmonary carcinoids with sensitivity of 96% and 100% specific while 18F-FDG PET/CT scan suffers from low sensitivity and specificity in differentiating the pulmonary carcinoids from other tumors [8].

Carcinoid tumors, when localized, are primarily treated surgically. With surgical removal the five-year survival is 97% and 78% for typical and atypical carcinoid tumors, respectively [2]. The histology and lymph node involvement were the main prognostic factors for these patients [2]. However, for patients with metastatic disease there is little to offer as metastatic tumors are generally not sensitive to chemotherapy or radiotherapy.

Bronchoscopic ablation is a useful and efficacious modality for the removal of endobronchial tumors [1, 4, 9]. The indications of endobronchial ablative therapy include an endobronchial tumor occupying more than 50% of the lumen of large airways associated with dyspnea and hemoptysis related to the lesion, lesions preventing the mucociliary clearance and causing recurrent pneumonitis or intractable cough. Only absolute contraindication of endobronchial ablative therapy is the extrinsic compression of the airways. Both flexible as well as rigid bronchoscope may be used for endobronchial ablation. In patients with significant airway obstruction and especially patients who are in respiratory failure, rigid bronchoscopy is the preferred modality for removal. The large lumen of the ventilating rigid bronchoscope allows ventilation to continue while airway procedures are being simultaneously performed. Removal of the endobronchial growths can be accomplished by either coring out the tumor using the beveled tip of the rigid bronchoscope or by application of endobronchial ablative modalities like laser, electrocautery, cryotherapy, and so forth [3, 9, 10]. Flexible bronchoscopic removal of endobronchial tumors can also be performed efficaciously especially in the hands of trained operators. We performed endobronchial removal, since CT scan was suggestive of isolated endobronchial lesion without extension through the cartilaginous area. However, it must be kept in mind that inadvertent complications may arise during flexible bronchoscopic removal like tumor displacement, significant bleeding, and so forth which might necessitate urgent rigid bronchoscopy.

## 4. Conclusion

Central airway tumors are mimickers of bronchial asthma and chest X-ray can be normal; hence, CT should be considered in patients who have symptoms of stridor or trepopnea. Tracheal carcinoid without extension to the mediastinum can be successfully removed using flexible bronchoscopy and electrocautery but needs close follow-up of the excision site for recurrence.
